# Workaholism, Work Engagement and Child Well-Being: A Test of the Spillover-Crossover Model

**DOI:** 10.3390/ijerph17176213

**Published:** 2020-08-27

**Authors:** Akihito Shimazu, Arnold B. Bakker, Evangelia Demerouti, Takeo Fujiwara, Noboru Iwata, Kyoko Shimada, Masaya Takahashi, Masahito Tokita, Izumi Watai, Norito Kawakami

**Affiliations:** 1 Department of Policy Management, Keio University, 5322 Endo, Fujisawa, Kanagawa 252-0882, Japan; 2 Center of Excellence for Positive Organizational Psychology, Erasmus University Rotterdam, P.O. Box 1738, 3000DR Rotterdam, The Netherlands; bakker@essb.eur.nl; 3 Department of Industrial Engineering & Innovation Sciences, Eindhoven University of Technology, P.O. Box 513, 5600MB Eindhoven, The Netherlands; E.Demerouti@tue.nl; 4 Department of Global Health Promotion, Tokyo Medical and Dental University, 1-5-45 Yushima, Bunkyo-ku, Tokyo 113-8519, Japan; fujiwara.hlth@tmd.ac.jp; 5 Department of Nursing, Kiryu University, 606 Azami, Kasakake-Cho, Midori, Gunma 379-2393, Japan; iwata-no@kiryu-u.ac.jp; 6 Institute of Social Sciences, Toyo University, 5-28-20, Hakusan, Bunkyo-ku, Tokyo 112-8606, Japan; kshima-tky@umin.ac.jp; 7 Research Center for Overwork-Related Disorders, National Institute of Occupational Safety and Health, 6-21-1, Nagao, Tama-ku, Kawasaki 214-8585, Japan; takaham@h.jniosh.johas.go.jp; 8 Graduate School of Media and Governance, Keio University, 5322 Endo, Fujisawa, Kanagawa 252-0882, Japan; tokitam-tky@umin.ac.jp; 9 Community Health Nursing, Hamamatsu University School of Medicine, 1-20-1, Handayama, Higashi-ku, Hamamatsu 431-3192, Japan; izumiw@hama-med.ac.jp; 10 Department of Mental Health, The University of Tokyo, 7-3-1 Hongo, Bunkyo-ku, Tokyo 113-0033, Japan; norito@m.u-tokyo.ac.jp

**Keywords:** happiness, spillover-crossover model, workaholism, work engagement, work-family balance

## Abstract

This study examines how working parents’ work attitudes (i.e., workaholism and work engagement) are associated with their child’s psychological well-being. Based on the Spillover-Crossover model (SCM), we hypothesize that (a) work-to-family spillover (i.e., work-to-family conflict and facilitation) and (b) employee happiness will sequentially mediate the relationship between parents’ work attitudes and their child’s emotional and behavioral problems. A cross-sectional survey was conducted among Japanese dual-earner couples with pre-school child(ren). On the basis of valid data from 208 families, the hypothesized model was tested using structural equation modeling. For both fathers and mothers simultaneously, workaholism was positively related to work-to-family conflict, which, in turn, was negatively related to happiness. In contrast, work engagement was positively related to work-to-family facilitation, which, in turn, was positively related to happiness. Fathers’ and mothers’ happiness, in turn, were negatively related to their child’s emotional and behavioral problems. Results suggest that parents’ workaholism and work engagement are related to their child’s emotional and behavioral problems in opposite ways, whereby parents’ spillover and happiness mediate this relationship. These findings support the SCM and suggest that decreasing workaholism and improving work engagement may not only improve employees’ happiness, but also decrease their child’s emotional and behavioral problems.

## 1. Introduction

Rapidly changing working conditions (e.g., global competition, high pace of innovation) stimulate employees to work harder and put more energy into work than before. Heavy work investment is characterized as a strong focus on the task at hand and a high level of dedication to work [1]. A broad literature on heavy work investment has studied the effects on one’s own and one partner’s well-being [2,3,4,5,6]. However, heavy work investment may also affect the well-being of one’s children [7].

Mental health problems are responsible for 8.5% of disability-adjusted life years among children aged 5–9 years old [8]. Raising a child with emotional and behavioral problems increases strain on parents [9]. If the parents have a paid job, they may also experience difficulties in the work domain (e.g., work-family imbalance) [10]. In light of increasing mental health problems among children in recent decades [11], more attention should be paid to the influence of parents’ heavy work investment on their children’s emotional and behavioral problems.

In the current study among Japanese couples, we will examine how parents’ heavy work investment is associated with their child’s emotional and behavioral problems. Linking the literature on work-family and positive psychology, we uncover the underlying mechanisms of how work attitudes of parents are associated with their child’s well-being using the Spillover-Crossover model (SCM) [12,13]. Following the distinction between different types of heavy work investment [14], we examine whether the two different types of heavy work investment—workaholism and work engagement—have antagonistic relationships with a child’s well-being. In addition, by using the data from dual-earner couples, we explore whether both parents influence their child’s well-being in similar ways.

In this way our study contributes to the literature on SCM by contrasting antagonistic relationships of two forms of heavy work investment with family life. Our study also contributes to the literature on child’s well-being by uncovering how heavy parental work investment is related to the child’s well-being both in a favorable and detrimental way.

### 1.1. The Spillover-Crossover Model

The SCM presents the two different ways in which experiences are carried over from the work to the family domain. Spillover is a within-person, across-domains transmission of demands and consequent strain from the work domain to the nonwork domain. This process is also called work-to-family conflict, referring to the interference of work with private life [6]. In contrast, crossover involves transmission across individuals, whereby demands and their consequent strain cross over between closely related persons [15]. The Spillover-Crossover model integrates both approaches. In her theoretical analyses, Westman [15,16] includes workaholism as a personal characteristic that may influence the crossover process. Previous studies revealed the spillover-crossover process from one partner’s workaholism to the other partner’s well-being through family-to-work conflict [3], relationship satisfaction [17], and family satisfaction [6].

Although previous work-family studies mainly focused on negative spillover, research has clearly indicated that positive spillover is also possible. Work-family facilitation is defined as “the extent to which participation at work (or home) is made easier by virtue of the experiences, skills, and opportunities gained or developed at home (or work)” [18] (p. 145). Bakker et al. [6] showed that two types of heavy work investment, workaholism and work engagement, were negatively and positively associated with one’s own and one’s partner’s family satisfaction through work-to-family conflict and work-to-family facilitation, respectively. These findings suggest that positive crossover is also possible. The present study expands previous research with the SCM by focusing on two different types of spillover simultaneously and examining the crossover from parents to their child’s well-being in one overall SCM.

### 1.2. Two Types of Heavy Work Investment: Workaholism and Work Engagement

Two types of heavy work investment can be distinguished, workaholism and work engagement [14]. Workaholism is defined as “a tendency to work excessively hard and to be obsessed with work, which manifests itself in working compulsively” [19] (p. 204). In contrast, work engagement is defined as “a positive, fulfilling, work-related state of mind that is characterized by vigor, dedication, and absorption” [20] (p. 74). Although both workaholism and work engagement are characterized by active and work-related states that are indicative of heavy work investment [21], the underlying motivation for this investment differs. Workaholics are propelled by an obsessive inner drive they cannot resist, whereas engaged employees are intrinsically motivated [2,22]. Put differently, workaholism is characterized by high effort with negative affect, whereas work engagement is characterized by high effort with positive affect [23].

This distinctiveness is theoretically linked to obsessive versus harmonious passion [24,25,26,27]. Obsessive passion results from an overcontrolled internalization of an activity into one’s identity and overwhelms one’s attention. In contrast, harmonious passion results from an autonomous internalization of an activity into one’s identity [28]. Activity with harmonious passion occupies a meaningful—but not overwhelming—place in one’s life and remains in harmony with other aspects of a person’s life [29].

The distinction between workaholism and work engagement was empirically demonstrated in terms of their relationship with various indicators of employees’ own well-being and job performance. For instance, workaholism is associated with (future) unwell-being (i.e., high ill-health and low job and family satisfaction) and poor job performance, whereas work engagement is associated with (future) well-being (i.e., low ill-health and high job and family satisfaction) and superior job performance [2,4,5]. Bakker et al. [6] expanded the literature by examining the influence of one’s workaholism and work engagement on the partner’s family satisfaction, suggesting crossover from one’s heavy work investment to the partner well-being. The current study further expands Bakker et al. [6] by examining the crossover to one’s child’s well-being.

### 1.3. The Current Study

**Figure 1:**
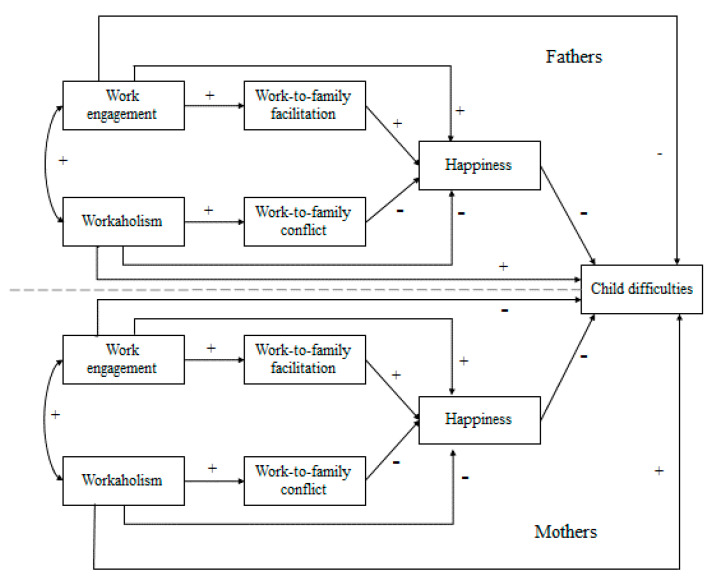
The hypothesized model in the present study. Note: + hypothesized relationship positive, —hypothesized relationship negative.

Figure 1 presents the model that will be tested in the current study.

We predict that workaholism will have a negative relationship with one’s own happiness, because workaholism increases the likelihood of work-to-family conflict. Work-to-family conflict occurs when role demands from the work domain are mutually incompatible with the role demands of the family domain leading to role strain [30]. Consequently, work-to-family conflict will make people unhappy because they are inhibited from investing time and energy in life domains where they have an active role. Individuals feel happy when they allocate their energies among these competing demands in line with their guiding goals and values. When they experience work-family conflict, family values and work values are competing factors (e.g., professional advancement vs. family investment of time) which hinders personal happiness and fulfillment [31]. Since workaholics invest by definition a lot of time at work leaving less time available for the family role, enhancing in this way work-to-family conflict, we predict:

**Hypotheses** **1** **(H1).** *Workaholism has a negative relationship with happiness, through work-to-family conflict.*

In contrast, we predict that work engagement will have a positive relationship with one’s own happiness, because work engagement increases the likelihood of work-to-family facilitation. Inter-role facilitation can occur in an instrumental pathway through skills and opportunities for self-growth and in an affective pathway by positive affect via positive emotions and levels of energy [32,33,34]. When these positive gains such as enhanced skills, opportunities for self-growth, and positive affect from one domain are applied, sustained and reinforced in another domain, the end result is improved system functioning or facilitation. The accumulation of resources inherent in work-to-family facilitation can result in enhanced outcomes within personal life. In this way the experience of work-to-family facilitation should enhance happiness (representing a positive affective state) [35]. Since engaged workers, compared to workaholics, show better performance and are likely to experience positive emotions [23], we predict:

**Hypotheses** **2** **(H2).** *Work engagement has a positive relationship with happiness, through work-to-family facilitation.*

Several previous studies have supported the SCM, showing that experiences in the work domain spill over to the home domain, and, consequently, cross over to the partner. However, few studies have explicitly investigated the link between parents’ experience in the work domain and their child’s well-being. Nevertheless, we can assume the crossover link in the following three pathways.

First is parenting behavior. According to the conceptual model of Armstrong [36], parental well-being is related to quality of parenting, which results in child well-being. Indeed, Howard Sharp et al. [37] showed that parental distress was related to poor parenting (i.e., less warmth, more psychological control, and more problematic communication), which resulted in child’s behavioral problems. Kaiser et al. [38] also showed that poor parental well-being (i.e., poverty) was associated with poor parenting (i.e., more psychological control), which resulted in the child’s emotional and behavioral problems. These findings suggest that parents’ poor well-being due to workaholism and work-to-family conflict is associated with their child’s emotional and behavioral problems through negative parenting behavior. Second is transmission of (positive) emotion from parents to child. According to emotional contagion theory [39], people catch other people’s emotions through automatic mimicry of the facial expressions, vocal expressions, postures, and instrumental behaviors, and feedback of other’s emotions. When parents are happy (representing a positive emotion), their child catches this emotion and consequently the child’s well-being is improved (i.e., lowered emotional and behavioral problems). Third is family functioning. Work-to-family conflict is related to decreased social support from and increased social undermining by the partner [40]. In addition, psychological distress caused by work-to-family conflict is related to hostile interactions and lowered marital warmth and supportiveness between the partners [41]. When parents are happy, with low psychological distress, their relationship quality is good, and the family creates a psychologically safe place. In a psychologically safe family, a child can have secure attachment and grow and experiment without fear of punishment. On the basis of these argumentations, we predict that:

**Hypotheses** **3** **(H3).** *Parents’ workaholism has a positive relationship with their child’s emotional and behavioral problems, through work-to-family conflict and their own happiness (sequential mediation).*

Finally, we expected that parents’ work engagement would have a negative indirect relationship with their child’s emotional and behavioral problems. Although few studies have explicitly investigated the favorable relationship of parents’ work engagement with their child’s well-being, we can still expect this relationship. Previous findings provide evidence for positive spillover (see [42] for a meta-analysis), whereby positive experiences at work can lead to one’s own well-being through work-to-family facilitation. Since both positive and negative work experiences (i.e., work engagement and workaholism, respectively) crossed over to partner [6], we can assume that positive experiences of parents are just as likely to cross over to their child as negative experiences through the three pathways mentioned above [43,44]. On the basis of these argumentations, we predict that:

**Hypotheses** **4** **(H4).** *Parents’ work engagement has a negative relationship with their child’s emotional and behavioral problems, through work-to-family facilitation and their own happiness (sequential mediation).*

Finally, we will explore whether the gender of the parent plays a moderating role in these relationships. Since there is insufficient evidence for differential relationships for mothers versus fathers, we do not pose specific hypotheses.

## 2. Materials and Methods

### 2.1. Procedure and Participants

This study is part of the Tokyo Work–Family Interface Study (TWIN) II, a large cohort study that commenced in 2011. The TWIN study aims at examining intra-individual (i.e., spillover) and inter-individual (i.e., crossover) processes of well-being among all dual-earner couples with preschool children in Tokyo, Japan. To the best of our knowledge, this is one of the largest work-family interface studies that collected data from dual-earner couples.

We invited participants of TWIN study I [45], the study we conducted in the Setagaya-ward, Tokyo, in 2008–2009, to participate in the TWIN II survey (*N* = 321 families). Additionally, we also approached dual-earner couples with preschool children in another ward, Meguro-ward, through all nursery schools (*N* = 22). After checking the signed consent forms from those parents who agreed to participate, we directly sent out questionnaires to participating parents’ addresses (*N* = 357 families). In total, questionnaires were distributed to 678 families in 2011. For more details about the procedure, please refer to Fujiwara et al. [46]. The whole procedure followed in the present study was reviewed and approved by the Ethics Committees of the Graduate School of Medicine at the University of Tokyo.

The researchers distributed three questionnaires to each family: two identical questionnaires for the parents (one for each partner), and one questionnaire for their child. The partners were kindly asked to fill out their questionnaires independently. Regarding the questionnaire for the child, one of the partners was asked to answer. If parents had two or more children, they were asked to answer the questions with the youngest child in mind. Respondents returned their questionnaires in closed, pre-stamped envelopes to a researcher at the University of Tokyo. Of the questionnaires distributed among 678 families, 413 were returned, resulting in a response rate of 60.9%. In this study, data on children with a reported age of <24 months was excluded, because the measure of child’s emotional and behavioral problem (i.e., The Strength and Difficulties Questionnaire: SDQ) can be applicable only for a child of ≥24 months [47]. Data on children with a reported age of ≥72 months were also excluded because they were considered to be elementary school students, not preschool children. In total, the matched responses of 208 families who answered all the three questionnaires were analyzed.

As can be seen in Table 1, fathers were almost two years older than mothers, *t* (207) = 5.21, *p* < 0.001. There were also differences between fathers and mothers regarding educational background, work hours, and the time spent for housework and child rearing and with child(ren) (*χ*^2^ (12) = 60.88, *p* < 0.001; *t* (201) = 9.82, *p* < 0.001, *t* (198) = −15.76, *p* < 0.001; *t* (203) = −11.31, *p* < 0.001). With regard to education background, the most frequently mentioned level of education for fathers was university (55.8%) followed by high school (23.6%) and graduate school (17.8), whereas that for mothers was university (53.4%) followed by junior college (18.3%) and high school (15.4%). With regard to working hours, fathers worked 14 h longer than mothers. In contrast, mothers spent more time on housework and child-rearing and with child(ren) than fathers. With regard to the number of children, most couples had one child (52.4%) or two children (36.1%). The mean age of the youngest child was 44.6 months. Of the children of these parents, 53.8% were male. Most of the questionnaires for children were answered by mothers (92.8%).

### 2.2. Measures

#### 2.2.1. Workaholism

Workaholism was measured with the Dutch Workaholism Scale (DUWAS) [21]. The scale consists of two subscales; Working Excessively (e.g., “I stay busy and keep many irons in the fire”) and Working Compulsively (e.g., “I feel guilty when I take time off work”). Each subscale consists of five items that were rated on a 4-point Likert scale (1 = totally disagree, 4 = totally agree). All items were summed to form one overall index of workaholism and averaged to get an average score, creating a possible range of 1–4.

#### 2.2.2. Work Engagement

Work engagement was measured with the nine-item version of the Utrecht Work Engagement Scale (UWES) [48,49]. The UWES reflects three underlying dimensions, which are measured with three items each: Vigor (e.g., “At my work, I feel bursting with energy”), Dedication (e.g., “My job inspires me”), and Absorption (e.g., “I get carried away when I am working”). High scores on all three dimensions indicate high work engagement. Items were scored on a scale ranging from (0) “never” to (6) “always”. The nine items were summed to form one overall index of work engagement and averaged to get an average score, creating a possible range of 0–6.

#### 2.2.3. Work-to-Family Conflict

Work-to-family conflict was measured with three items from the Survey Work-home Interaction-NijmeGen (SWING) [50,51]. An example item is “How often does it happen to you that your work schedule makes it difficult for you to fulfill your domestic obligations?” (0 = never, 3 = always). Responses for the three items were summed and averaged to get an average score, creating a possible range of 0–3.

#### 2.2.4. Work-to-Family Facilitation

Work-to-family facilitation was assessed with four items from the SWING [50,51]. An example item is “How often does it happen to you that you manage your time at home more efficiently as a result of the way you do your job?” (0 = never, 3 = always). Responses for the four items were summed and averaged to get an average score, creating a possible range of 0–3.

#### 2.2.5. Happiness

Happiness was measured with a single item. Respondents were asked to rate their overall happiness on a 0–10 self-anchoring scale, in which 0 is defined as the “not happy at all” and 10 is defined as “very happy”. A one-item happiness scale is often used in happiness research [52].

#### 2.2.6. Child’s Emotional and Behavioral Problems

Child’s emotional and behavioral problems were assessed by the parents with the Japanese version of The Strength and Difficulties Questionnaire (SDQ) [47,53]. SDQ assesses the child’s emotional distress (e.g., often unhappy, downhearted or tearful), conduct and oppositional behaviors (e.g., often has temper tantrums or hot tempers), hyper activity and inattention (e.g., restless, overactive, cannot stay still for long) and peer problems (e.g., picked on or bullied by other children). Based on the parent ratings (response categories 0 = not true, to 2 = certainly true), a total child difficulties scores was formed by summing the 20 items and averaged to get an average score, creating a possible range of 0–2.

Note that all Cronbach’s alpha coefficients are reported in Table 2.

### 2.3. Strategy of Analysis

Our data were analyzed by means of structural equation modeling (SEM) techniques using the AMOS software package version 26. For the analysis of dyadic data, SEM and multilevel modeling (MLM) are the dominant methods, and each method has its own advantages and disadvantages [54]. Using SEM is especially straightforward in the analysis of mediation within the dyadic nature of the data [55] because it allows the estimation of the full model in one run. Therefore, we adopted SEM in our analyses. In order to avoid overly complex models, all model variables were included as manifest, observed variables, i.e., the mean score of each variable [56].

To test our hypotheses, we examined whether individual paths as well as indirect relationships were significant. We followed the recommendations of Aguinis et al. [57] for testing mediation effects; namely, we conducted the mediation test without the precondition that the relationship between the predictor and the outcome should be significant. We computed the size of the indirect effect by multiplying the paths to and from the mediator, and tested this product using the bootstrap method. Please note that we also added the direct paths from workaholism and work engagement to one’s happiness and to the child’s emotional and behavioral problems, because our study does not include all possible mediators of the processes under study. In addition, we added two covariances between workaholism and work engagement in line with previous studies (e.g., [2,4,5,6]) and between the father’s happiness and mother’s happiness according to crossover theory [16].

Next to the chi-square (χ^2^) statistic we inspected the Goodness of Fit Index (GFI) and the Root Mean Square Error of Approximation (RMSEA). In addition, three fit indices that are less sensitive to sample size were used: the Comparative Fit Index (CFI), the Incremental Fit Index (IFI), and the Tucker-Lewis Index (TLI). For each of these statistics, values of 0.90 are acceptable and values of 0.95 or higher are indicative of good fit [58], except for the RMSEA for which values of 0.05 indicate good fit and values up to 0.08 represent reasonable errors of approximation [59].

## 3. Results

### 3.1. Descriptive Statistics

Table 2 shows the means, standard deviations, correlations, and the internal consistencies of all scales included in this study. As can be seen, the reliabilities were acceptable for all scales except for child’s emotional and behavioral problems (i.e., Difficulties). Although one’s own workaholism and work engagement are unrelated to those of the partner, fathers’ and mothers’ happiness are related. For both fathers and mothers, workaholism is related to work-to-family conflict (WFC) while work engagement is related to work-to-family facilitation (WFF). In addition, one’s own WFC and WFF are related to one’s own happiness for both fathers and mothers. Furthermore, fathers’ and mothers’ happiness are related to their child’s emotional and behavioral problems.

### 3.2. Test of the Spillover-Crossover Model

**Figure 2:**
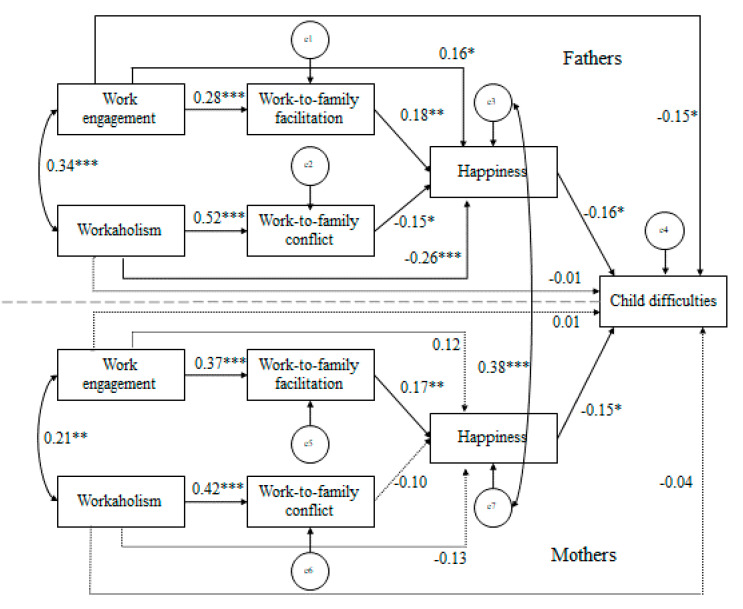
Standardized solution for the hypothesized model (*N* = 208 families). Note: *** *p* < 0.001, ** *p* < 0.01, * *p* < 0.05. Dotted lines represent nonsignificant paths (*p* > 0.05).

Figure 2 shows the results of the hypothesized model. The hypothesized model showed a good fit to the data: (*χ*^2^ = 53.91, *df* = 34, *p* = 0.016, GFI = 0.96, RMSEA = 0.05, CFI = 0.94, IFI = 0.94). For both fathers and mothers, workaholism was positively related to WFC, which was, in turn, negatively related to one’s own happiness. The bootstrap method indicated that this indirect relationship was significant for fathers (−0.078; 95% CI between −0.036 and −0.005, *p* = 0.025), but not for mothers (−0.042; 95% CI between −0.026 and 0.003, *p* = 0.174). This provides support for Hypothesis 1 only among fathers. Similarly, for both fathers and mothers work engagement was positively related to WFF, which, in its turn, was positively related with one’s own happiness. The bootstrap method indicated that this indirect relationship was significant for fathers (0.051; 95% CI between 0.002 and 0.014, *p* = 0.007), and for mothers (0.064; 95% CI between 0.004 and 0.022, *p* = 0.007). Thus, Hypothesis 2 was confirmed for fathers and mothers.

As for parents’ investment in hard work and child’s emotional and behavioral problems, we continued to examine the indirect relationship of parents’ workaholism with their child’s emotional and behavioral problems via WFC and their own happiness (cf. Hypothesis 3), and the indirect relationship of parents’ work engagement with their child’s emotional and behavioral problems via WFF and their own happiness (cf. Hypothesis 4). For both fathers and mothers, one’s own happiness was negatively related to the child’s emotional and behavioral problems. The bootstrap method indicated that the indirect relationship of fathers’ workaholism with their child’s emotional and behavioral problems was significant (0.052; 95% CI between 0.005 and 0.075, *p* = 0.049) whereas that of mothers’ workaholism was not significant (0.026; 95% CI between 0.001 and 0.040, *p* = 0.080). This provides support for Hypothesis 3 only among fathers. The bootstrap method indicated that the indirect relationship of fathers’ work engagement with their child’s emotional and behavioral problems was marginally significant for fathers (−0.032; 95% CI between −0.029 and −0.001, *p* = 0.051) and non-significant for mothers (−0.028; 95% CI between −0.027 and −0.001, *p* = 0.077). This does not provide support for Hypothesis 4 among fathers or mothers.

In the proposed model, we found additional significant direct pathways from workaholism and work engagement to happiness, and from work engagement to child’s emotional and behavioral problems only among fathers. In addition, we found a positive relationship between fathers’ and mothers’ happiness, suggesting crossover of happiness between fathers and mothers. Testing statistical differences between similar parameter estimates, we did not find any gender differences in the strength of the relationships in the model with all *t* values lower than 1.95. This suggests that gender did not affect the strength of the relationship in the hypothesized SCM.

When we conducted additional analyses to control for age and gender of the child and the rater of the child questionnaire (father or mother) as potential confounders, the path coefficients remained virtually the same as those of the model in Figure 2, but the model fit decreased slightly (*χ*^2^ = 96.94, *df* = 67, *p* = 0.010, GFI = 0.94, RMSEA = 0.05, CFI = 0.91, IFI = 0.91). These results indicate that the impact of the control variables on the model variables were weak. Importantly, none of the control variables affected the structural paths in the model (*p*’s > 0.05).

## 4. Discussion

The current study examined how Japanese parents’ heavy work investment is associated with their child’s psychological well-being. To our knowledge, this is the first study to examine the relationship between parents’ heavy work investment—workaholism and work engagement—with the child’s psychological well-being. We hypothesized that parents’ workaholism would have unfavorable, and that their work engagement would have favorable consequences for their own and their child’s well-being.

The current findings integrate and expand the literature on heavy work investment, work-family balance, positive psychology, and child well-being in several respects. First, we expanded the SCM by including two different types of heavy work investment (i.e., workaholism and work engagement) as starters of the model. Second, we included positive as well as negative spillover (i.e., WFF, WFC) and cross over to expand the SCM. Third, we examined the relationship of parents’ heavy work investment with their child’s well-being (i.e., emotional and behavioral problems) as well as their own well-being (i.e., happiness). Fourth, we explored the gender differences in the hypothesized relationship based on two sources of information (i.e., fathers and mothers).

### 4.1. Contributions

In general, the results of structural equation modeling analyses supported our hypotheses. We found that parents’ workaholism and work engagement were oppositely related to their own happiness through WFC and WFF, respectively. The results revealed the underlying mechanisms of how work-related experiences may spill over from the work domain to the family domain. Because workaholics, who work excessively hard and in a compulsive manner, tend to invest more resources in their work at the expense of other important life roles [60], they are more likely to experience WFC due to having fewer resources remaining to devote to their family [61]. In contrast, engaged workers are characterized by vigor, dedication and absorption, and are intrinsically motivated [20]. Thus, they show better performance and are likely to experience positive emotions [23] and consequently WFF and happiness.

Perhaps the most important theoretical contribution of this study is that it demonstrated the relationship of heavy work investment with the couple’s child’s emotional and behavioral problems by expanding the SCM. While previous research suggests the link between parents’ experience in the work domain and their child’s well-being, little research has directly examined the association. Our results showed that parents’ workaholism and work engagement had indirect relationships with their child’s emotional and behavioral problems through their own spillover and happiness in opposing ways. Although we are not able to determine the exact mechanisms of the association from our results, some explanations might be possible. Parenting behavior (e.g., psychological control, inconsistent parenting, harsh parenting, warmth withdrawal: [36,37,38]), transmission of affect (i.e., emotional contagion; [39]), and family functioning, such as a psychologically safe family, may mediate the relationship between parents’ happiness and their child’s emotional and behavioral problems. Future research is needed to clarify more detailed mechanisms.

Our findings also demonstrated that there exist two unique spillover-crossover pathways, starting with workaholism and work engagement [6]. Although workaholism and work engagement had weak but positive relationships for both genders, the consequences were opposite [2,4,5,6]. This suggests that workaholism and work engagement are two conceptually different types of heavy work investment. Although both concepts are characterized by active and work-related states that are indicative of heavy work investment [21], the underlying motivation for this investment differs. Workaholics are propelled by an obsessive inner drive they cannot resist, whereas engaged employees are intrinsically motivated [2,22]. This is in line with the theory of passion: obsessive versus harmonious passion [24,25,26,27]. According to Vallerand et al. [26,27], obsessive passion results from an overcontrolled internalization of an activity into one’s identity and overwhelms one’s attention, whereas harmonious passion results from an autonomous internalization of an activity into one’s identity. Thus, we can assume that workaholic employees with an obsessive passion are more likely to have adverse relationships with their own and their child’s well-being, whereas engaged employees with harmonious passion are more likely to have favorable relationships with their child.

It is worth noting gender differences in the link between parents’ experience in the work domain and their child’s well-being. We did not find any gender differences in the strength of the relationships in the model, suggesting that gender did not affect the strength of the relationships in the hypothesized SCM. This finding is in line with previous studies conducted in western societies (e.g., [12,62]). However, our results found additional significant direct pathways from workaholism and work engagement to happiness, and from work engagement to child’s psychological well-being only among fathers. This suggests that other mechanisms than hypothesized SCM can also be possible only among fathers. This discrepancy may reflect gender role differences, at least among Japanese couples. In Japan, mothers play a more important role in housework and child-rearing than fathers [63]. Indeed, our data showed that mothers spent more time on housework and child-rearing compared to fathers (43.8 h/week for mothers and 18.9 for fathers). Because mothers spent more time and energy on housework and child-rearing, a pathway from work experiences to happiness and child’s well-being mediated by WFC and WFF is more likely among mothers. In contrast, fathers spent more time on work-related activities compared to mothers (50.6 for fathers and 36.9 for mothers h/week). Thus, direct pathways from workaholism and work engagement to happiness, and from work engagement to child’s psychological well-being may be found only among fathers. This speculation can be explained by identity theory [64,65], which claims that people devote considerable time and energy to constructing and maintaining desired identities and that people feel threatened when their self-images are damaged by impediments to self-identifying activities.

The possible mechanisms of how fathers’ work engagement directly decreases their child’s emotional and behavioral problems require further research. Given nonsignificant correlations between fathers’ work engagement and time spent with child (r = −0.025) and between fathers’ time spent with child and their child’s emotional and behavioral problems (r = 0.037), time spent with child did not seem to mediate the association. Thus, the quality rather than the quantity of time spent might be more important (see also [46]). For example, fathers with higher work engagement might be more likely to spend time with their child with more warm attitudes, because they are characterized by positive, high-activated affect [23]. Further research is needed to investigate the association between parental experiences in the work domain and the quality of the time spent with child to elucidate the relationship between parental work engagement and child emotional and behavioral problems.

### 4.2. Strengths and Limitations

Our research has several strengths in terms of study design including matching data on father, mother and child, and theoretical expansion of heavy work investment, work-family balance, positive psychology, and child well-being. However, this study also has several limitations.

First, this study did not examine the underlying mechanisms between parents’ well-being (i.e., happiness) and their child’s well-being (i.e., emotional and behavioral problems). Previous research suggests that the relationship between parent well-being and child well-being can be mediated by the quality of the parent-child relationship [66] and by parenting style [36,37,38]. Given the direct relationship between parental work engagement and child’s emotional and behavioral problems, alternative mechanisms in the relationship between parents’ experience in the work domain and their child’s well-being should be tested. Second, the sample size of this study was relatively small. More studies with larger sample sizes are needed to validate our findings. Third, findings are based on a cross-sectional design. Due to the limited number of cases appropriate for longitudinal analysis, a causal relationship between parents’ workaholism and work engagement to their child’s emotional and behavioral problems could not be examined. Reversed causal relationships between child’s emotional and behavioral problems to parents’ happiness and work well-being (e.g., [10]), or reciprocal relationships, need to be tested in future research. Fourth, our respondents were all Japanese dual-earner couples with preschool children who lived in Tokyo. Further research is needed to determine whether we can generalize our findings to other areas and other (Western) countries. Finally, because parents (typically mothers) were the sole informants of child’s emotional and behavioral problems, the data may have contained same-source bias [67]. For example, parents’ psychological well-being may have influenced their ratings of the child’s emotional and behavioral problems. In addition, parents with heavy work investment (typically workaholism) may have had limited opportunities to observe their child’s psychological well-being. Future research should examine the relationship between parents’ work attitudes and their child’s psychological well-being using informants other than parents (e.g., preschool staff or teachers).

## 5. Conclusions

Two different types of employee work attitudes (i.e., workaholism and work engagement) were oppositely related to child’s emotional and behavioral problems through work-to-family conflict and work-to-family facilitation and happiness. These findings support the SCM and suggest that decreasing workaholism and improving work engagement may not only improve employees’ own happiness, but also decrease their child’s emotional and behavioral problems.

## Figures and Tables

**Table 1:** Comparison of Means (and SDs) or numbers (and percentages) of demographic and variables between Fathers and Mothers.

	Fathers				Mothers				Statistical Test	*p* Value
	*n ^a^*	Mean	(*SD*)	(%)	*n*	Mean	(*SD*)	(%)		
Parents										
Age	208	39.7	(6.0)		208	38.1	(4.2)		*t* (207) = 5.21 ^b^	<0.001
Education										
Junior high school	2			(1.0)	0			(0.0)	*χ*^2^ (12) = 60.88	<0.001
High school	49			(23.6)	32			(15.4)		
Junior college	4			(1.9)	38			(18.3)		
University	116			(55.8)	111			(53.4)		
Graduate school	37			(17.8)	27			(13.0)		
Occupational classification									*χ*^2^ (40) = 51.24	>0.05
Professional or technician	80			(39.0)	68			(33.2)		
Managerial workers	43			(21.0)	9			(4.4)		
Clerical workers	22			(10.7)	82			(40.0)		
Sales worker	26			(12.7)	11			(5.4)		
Service worker	11			(5.4)	15			(7.3)		
Manufacturing process workers	6			(2.9)	0			(0.0)		
Security workers	2			(1.0)	1			(0.5)		
Agriculture, forestry, and fishery workers	0			(0.0)	0			(0.0)		
Transport and postal activity workers	4			(2.0)	0			(0.0)		
Others	11			(5.4)	19			(9.3)		
Work hours (per week)	202	50.6	(13.6)		202	36.9	(12.9)		*t* (201) = 9.82 ^b^	<0.001
Time spent for housework & child-rearing (hours per week)	199	18.9	(13.3)		199	43.8	(17.5)		*t* (198) = −15.76 ^b^	<0.001
Time spent with child(ren) (hours per week)	204	27.9	(19.6)		204	53.0	(25.5)		*t* (203) = −11.31 ^b^	<0.001
Child										
Number of child(ren)										
1	109			(52.4)						
2	75			(36.1)						
3	19			(9.1)						
4	5			(2.4)						
Age (months)	208	44.6	(13.2)							
Gender										
Male	112			(53.8)						
Female	96			(46.2)						
Rater										
Mother	193			(92.8)						
Father	15			(7.2)						

^a^ The numbers did not add up to the total number of the participants because of occational missing data. ^b^ Paired *t*-test.

**Table 2:** Means, SDs, Cronbach’s Alphas, and correlations of the variables used in the study (*N* = 208 families).

	Measures	Mean	SD	1	2	3	4	5	6	7	8	9	10	11
*Fathers (n = 208)*														
1	Workaholism	2.18	0.57	(0.82)										
2	Work engagement	3.14	1.11	0.34 ***	(0.93)									
3	WFC	0.71	0.60	0.52 ***	0.08	(0.73)								
4	WFF	1.38	0.66	0.21 **	0.28 ***	0.03	(0.71)							
5	Happiness	7.84	1.51	−0.25 ***	0.14 *	−0.31 ***	0.20 **							
*Mothers (n = 208)*														
6	Workaholism	2.07	0.58	0.08	0.17 *	0.06	0.10	0.15 *	(0.81)					
7	Work engagement	3.21	0.95	0.07	0.06	0.01	0.09	−0.02	0.21 **	(0.90)				
8	WFC	0.65	0.57	0.00	0.07	0.08	0.01	−0.08	0.42 ***	0.05	(0.70)			
9	WFF	1.46	0.75	0.05	0.05	−0.09	0.22	0.09	0.07	0.37 ***	0.00	(0.79)		
10	Happiness	7.91	1.64	−0.04	0.07	−0.16 *	0.14	0.40 ***	−0.08	0.14 *	−0.18 *	0.23 ***		
*Child (n = 208)*														
11	Difficulties	0.43	0.20	−0.01	−0.19 **	0.08	−0.04	−0.24 ***	−0.07	−0.03	0.02	−0.15 *	−0.22 **	(0.67)

Note: *** *p* < 0.001, ** *p* < 0.01, * *p* < 0.05. Alpha coefficients are displayed in parentheses.
